# Intelligent *L*2-*L*∞ Consensus of Multiagent Systems under Switching Topologies via Fuzzy Deep *Q* Learning

**DOI:** 10.1155/2022/4105546

**Published:** 2022-02-16

**Authors:** Haoyu Cheng, Linpeng Xu, Ruijia Song, Yue Zhu, Yangwang Fang

**Affiliations:** ^1^Unmanned System Research Institute, Northwestern Polytechnical University, Xi'an, China; ^2^School of Marine and Technology, Northwestern Polytechnical University, Xi'an, China; ^3^China Airborne Missile Academy, Luoyang 471009, China; ^4^School of Astronautics, Northwestern Polytechnical University, Xi'an, China; ^5^Beijing Institute of Remote Sensing Equipment, Beijing, China

## Abstract

The problem of intelligent *L*_2_-*L*_∞_ consensus design for leader-followers multiagent systems (MASs) under switching topologies is investigated based on switched control theory and fuzzy deep *Q* learning. It is supposed that the communication topologies are time-varying, and the model of MASs under switching topologies is constructed based on switched systems. By employing linear transformation, the problem of consensus of MASs is converted into the issue of *L*_2_-*L*_∞_ control. The consensus protocol is composed of the dynamics-based protocol and learning-based protocol, where the robust control theory and deep *Q* learning are applied for the two parts to guarantee the prescribed performance and improve the transient performance. The multiple Lyapunov function (MLF) method and mode-dependent average dwell time (MDADT) method are combined to give the scheduling interval, which ensures stability and prescribed attenuation performance. The sufficient existing conditions of consensus protocol are given, and the solutions of the dynamics-based protocol are derived based on linear matrix inequalities (LMIs). Then, the online design of the learning-based protocol is formulated as a Markov decision process, where the fuzzy deep *Q* learning is utilized to compensate for the uncertainties and achieve optimal performance. The variation of the learning-based protocol is modeled as the external compensation on the dynamics-based protocol. Therefore, the convergence of the proposed protocol can be guaranteed by employing the nonfragile control theory. In the end, a numerical example is given to validate the effectiveness and superiority of the proposed method.

## 1. Introduction

In recent years, the coordination control of MASs has attracted considerable attention for their broad applications in many fields [1, 2], such as formation control, cooperative attack, and attitude alignment. The MAS consists of a series of agents, which can communicate and interact with each other to realize multiple missions and adapt to the complex environment [3, 4]. In particular, much attention has been paid to the problem of consensus of MASs because of their great potential applications in both economic and military. The purpose of MASs is to construct a relationship between the agents to achieve an agreement for the state/output. In the past decades, fruitful research studies have emerged to contribute to the development in theory and applications. To mention a few, the problem of distributed formation control for MASs is studied in [5], the time-varying formation design for MASs with disturbances is proposed in [6], and the problem of finite-time consensus for switched nonlinear MASs is investigated in [7].

In practical applications, it is well known that the communication topology among the agents may change dramatically over time to adjust to multiple missions and complex environments [8, 9], such as the MASs can realize obstacle avoidance and higher flight efficiency by formation transformation [10, 11]. The design flexibility, security, and performance of convergence will be improved, which motivated the studies on the switching topologies of MASs [1, 12]. Recently, because of the broad potential applications of switching topologies, considerable significant research studies have been proposed by scholar at home and abroad. The communication topologies among interacting agents will change according to the flight conditions and missions, which can be modeled as switched systems. The switched systems consist of a series of continuous-time (or discrete-time) subsystems and a switching signal, which determines the switching strategy between subsystems. It provides an efficient approach to deal with the problem of fast time-varying conditions. Therefore, it can be inferred that the switching of topologies can be viewed as the switching between subsystems, and it is essential to study the problem of consensus protocol design to make sure the state/output can converge to the given value. In [13], the problem of time-varying formation control of MASs is investigated. The communication topologies switching among given connected topologies and the switching signal depend on the Markovian process. The Lyapunov function method is utilized to analyze the convergence. In the work of [14], the problem of event-triggered leader-following consensus problem for multiagent systems with external disturbances is addressed under switching topologies. A novel distributed event-triggered protocol is proposed to realize disturbance rejection based on extended state observer. The average dwell time (ADT) method is utilized to ensure the stability of the event-triggered protocol. In [15], the time-varying practical formation problem is studied for spacecraft, where switching topologies and time-delays are taken into consideration. Sufficient conditions are provided to ensure that the error system is convergent, which are derived based on the ADT method. It is well known that the research studies mentioned above are proposed to deal with the problem of switching topologies. However, the convergence is guaranteed based on the ADT method. It can be inferred that the common parameters are applied for all subsystems in the ADT method, which will lead to conservativeness. To obtain tighter bounds on dwell time and improve the design flexibility of the algorithm, MDADT is applied during last decades. In [16], the MDADT method and multiple discontinuous Lyapunov function (MDLF) method are combined to analyze the stability of switched systems with unstable modes. The sufficient conditions are established, and the results in existing literature are covered as a special case. The fast switching and slow switching in the framework of MDADT are applied to unstable modes and stable modes. In [17], the global adaptive control algorithm for switched systems is proposed based on the MDADT method. The different properties of subsystems are taken into consideration. Then, the adaptive tracking controller is applied to the nonlinear switched systems with external disturbance and unmodeled dynamics, which illustrates the effectiveness and superiority of the MDADT method. In the work of [18], the event-triggered sliding mode controller is proposed. By employing the MDADT method and event-triggered strategy, less conservative and more practical results are obtained. Sufficient conditions are given to ensure stochastically exponential stability by the aid of the LMI technique. The literature mentioned has provided fruitful results on consensus protocol design for MASs under switching topologies. However, stability and convergence are ensured by the traditional ADT method. The different properties of subsystems cannot be considered, which will lead to conservativeness. Therefore, how to obtain less restrictive results is still an open and challenging problem, which has been fully investigated, and it has an important value and potential applications in practice.

Moreover, in practical environment, there always exist uncertainties and disturbances, which will lead to performance degradation and even instability [19, 20]. Therefore, it is essential to investigate the robust consensus problem to improve the performance in the uncertain environment [21–23]. In the work of [24], the problem of distributed H∞ containment control for MASs with switching topologies is studied. An observer-based containment control scheme is proposed. The external disturbance and time delay in the environment are taken into consideration, which is more applicable than the traditional method. By employing the Lyapunov function method and LMIs technique, the sufficient existing conditions and solutions of control protocol are given in the form of LMIs. In [25], the problem of time-varying formation of second-order discrete-time MASs under switching topologies and the time delay is investigated. The sufficient conditions are given to ensure MASs accomplish the mission of time-varying formation based on the state transformation method. The time delay and uncertainties are considered. Compared with the existing literature, the proposed can overcome the undesirable response caused by time delay and improve the transient performance. In the work of [26], the problem of formation control for tail-sitters in flight mode transitions is studied. The nonlinear dynamics and uncertainties are considered, and the robust time-varying formation control protocol is proposed. It is proven that the tracking errors can converge to the origin in finite time. The problem of *L*_2_-gain robust protocol for time-varying output formation-containment of MASs is addressed in [27]. The PID-based output-feedback control protocol is provided to ensure that all followers can track a time-varying formation reference, where communication delays and external disturbance are taken into consideration. The asymptotic stability of MASs is proved by the Lyapunov function method. However, as well known, the transient performance and robustness cannot achieve simultaneously. Therefore, we need to make comprise of the transient performance and robustness, which still remains an open and challenging problem.

In addition, with the development of computing ability, the intelligent technique has been an attractable problem during the last decades [28–30]. It is widely applied in the areas of target recognition, machine vision, robotic systems, and controller design [31, 32]. It provides an efficient method to improve the autonomy and design flexibility of the system [33]. The most widely used methods are the deep learning and reinforcement learning. As a combination of deep learning and reinforcement learning, the advantages of deep learning and reinforcement learning are adopted, which include the characteristics of self-fitting and self-learning. In the work of [34], the automatic completion of multiple peg-in-hole assemble tasks is realized. Because the traditional method requires an accurate contact model and complex analysis, the intelligent control method is formulated by constructing the task as a Markov decision process. The deep deterministic policy gradient (DDPG) algorithm is proposed to accomplish the task to achieve optimal policy and avoid risky actions. In [35], a noninteger PID controller is proposed based on the DDPG algorithm. The measurement noises and external disturbances are taken into consideration. The kinematic controller and dynamic controller are proposed to achieve optimal performance. The DDPG algorithm is given to compensate for the uncertainties and disturbances in the framework of actor-critic. A numerical example is given to illustrate the effectiveness of the proposed method. Cheng et al. [36] proposed the real-time controller for the problem of fuel-optimal moon landing. Because the traditional method cannot meet the demand of high requirements of real-time performance and autonomy, the deep reinforcement learning algorithm is proposed for the real-time optimal control based on actor-indirect method architecture. The deep neural networks are applied for initial guesses, and the efficiency of training data is guaranteed. The literature mentioned above has provided considerable meaningful results in the area of machine learning. However, to the best of the authors' knowledge, the intelligent consensus design for MASs with considerations of stability, robustness, and optimal transient performance has not been fully studied yet. It is essential and important to achieve optimal comprise of robustness and transient performance.

Based on the statement above, it can be inferred that the problem of the improvement of autonomy and design flexibility for the system needs to be studied. The problem of consensus protocol design for MASs under switching topologies has not been fully investigated yet. The design flexibility can be improved by employing tighter bounds on dwell time because less conservative results can be obtained, and it leaves more room to ensure the switching logic stays in the subsystems with better performance for long enough time. Moreover, it is of great importance to combine the advantages of the traditional method and intelligent technique, which can ensure convergence, robustness, and transient performance simultaneously. Therefore, the problem of intelligent *L*_2_-*L*_∞_ consensus design of MASs under switching topologies is investigated. The convergence and robustness are guaranteed by the Lyapunov function method and the MDADT method, which are more applicable. The transient performance is improved by fuzzy deep *Q* learning, in which the fuzzy reward function is proposed for the complex scheduling process. The main contributions of this study can be summarized as follows:

1. The *L*_2_-*L*_∞_ consensus protocol of MASs under switching topologies is designed. The problem of *L*_2_-*L*_∞_ consensus of MASs is converted into the problem of stability analysis for switched systems, which is more applicable than the traditional method. The MDADT method and multiple Lyapunov function method are combined to guarantee the stability and prescribed attenuation performance index, which can obtain tighter bounds on dwell time and less conservative results.
2. The consensus protocol is composed of the dynamics-based consensus protocol and learning-based consensus protocol. Compared with the traditional method, the proposed strategy can ensure the stability, robustness, and transient performance simultaneously.
3. The fuzzy reward function is utilized to improve the efficiency of the deep reinforcement learning algorithm. The design of reward function for the traditional method mainly depends on the experience of designer, which will lead to complexity. The fuzzy reward function can improve the data efficiency and ensure optimal performance.

The rest of the study is organized as follows: the preliminaries and problem statement are provided in Section 2; in Section 3, the main results of the study are given; the numerical example is given in Section 4, which is followed by the conclusion in Section 5.

## 2. Preliminaries and Problem Statement

In this study, it is supposed that MASs are composed of a leader labelled as 0 and *n* followers labelled as 1, 2,…, *n*. The connection topology among *n* followers can be described as a time-varying model with *N*_*f*_ topologies. We define *𝒢*_*σ*(*k*)_=(*𝒢*_1_, *𝒢*_2_,…, *𝒢*_*N*_*f*__) as undirected connected graph, respectively. ℋ=(1,2,…, *n*), *n* > 1 represents the set of finite nodes. *s*=*σ*(*k*) : [0, *∞*)⟶*R*={1,2,…, *N*_*f*_} denotes the switching signal, which is a piecewise continuous function of time and takes value in the finite set ℋ. *𝒜*_*σ*(*k*)_=(*a*_*ij*_^*σ*(*k*)^)_*n*×*n*_ and **ℒ**_*σ*(*k*)_=(*l*_*ij*_^*σ*(*k*)^)_*n*×*n*_ are the adjacency matrices of the undirected graph *𝒢*_*σ*(*k*)_ and the Laplacian matrix at time instant *k*, where *a*_*ij*_^*σ*(*k*)^ stands for the element of adjacency matrix, where *a*_*ij*_^*σ*(*k*)^=1 represents that the node *i* can obtain information from node *j*, and *l*_*ij*_^*σ*(*k*)^ is defined in the following equation.

(1) $$\begin{array}{c} \left\{\begin{array}{c} l_{ij}^{\sigma \left(k\right)}=-a_{ij}^{\sigma \left(k\right)}\;i\neq j,\\ l_{ii}^{\sigma \left(k\right)}=\sum_{j=1}^{n}a_{ij}^{\sigma \left(k\right)}\;i=j. \end{array}\right) \end{array}$$

Then, for given node *i* ∈ ℋ, we can define the neighbors of node *i* as *𝒩*_*i*,*σ*(*k*)_={*j* ∈ ℋ : *a*_*ij*_^*σ*(*k*)^=1}.

Another undirected connected graph is defined as ${\tilde{𝒢}}_{\sigma \left(k\right)}=\left({\tilde{𝒢}}_{1},{\tilde{𝒢}}_{2},\ldots ,{\tilde{𝒢}}_{N_{f}}\right)$ to indicate the information transformation between the leader and the followers with *n* nodes. Define a diagonal matrix Θ_*σ*(*k*)_=diag{*θ*_1_^*σ*(*k*)^, *θ*_2_^*σ*(*k*)^,…, *θ*_*n*_^*σ*(*k*)^}, where *θ*_*i*_^*σ*(*k*)^=1 stands for that the node *i* ∈ ℋ can obtain information from the leader; otherwise, we define *θ*_*i*_^*σ*(*k*)^=0.

Therefore, MASs with leader-followers can be described as in the following equations:

(2) $$\begin{array}{c} x_{0}\left(k+1\right)=Ax_{0}\left(k\right), \end{array}$$

(3) $$\begin{array}{c} \left\{\begin{array}{c} x_{i}\left(k+1\right)=Ax_{i}\left(k\right)+Bu_{i}\left(k\right)+D\omega_{i}\left(k\right)\\ z_{i}\left(k\right)=C\upsilon_{i}\left(k\right) \end{array}\right),i\in ℋ, \end{array}$$

where A, B, C, and D are the system matrices with appropriate dimensions, x_0_(*k*)=[x_01_(*k*), x_02_(*k*),…,x_0*p*_(*k*)]^*T*^ ∈ R^*p*^ represents the state vector of leader, x_*i*_(*k*)=[x_*i*1_(*k*), x_*i*2_(*k*), ..., x_*ip*_(*k*)]^*T*^ ∈ R^*p*^ is the state of the *i*^th^ follower, *u*_*i*_(*k*)=[u_*i*1_(*k*), u_*i*2_(*k*),…,u_*il*_(*k*)]^*T*^ ∈ R^*l*^ is the input of the *i*^th^ follower, z_*i*_(*k*)=[z_*i*1_(*k*), z_*i*2_(*k*),…,z_*iq*_(*k*)]^*T*^ ∈ R^*q*^ stands for the output of the *i*^th^ follower, and *ω*_*i*_(*k*) ∈ R^*m*^ denotes the external disturbance belonging to *L*_2_[0, *∞*). It is supposed that the agent *i* can obtain information from its neighbors and leader. Therefore, we define *υ*_*i*_(*k*) as relative state measurements of the *i*^th^ agent, which can be described as follows:

(4) $$\begin{array}{c} \upsilon_{i}\left(k\right)=\sum_{j\in N_{i,\sigma \left(k\right)}}\left({\text{x}}_{i}\left(k\right)-{\text{x}}_{j}\left(k\right)\right)+\theta_{i}^{\sigma \left(k\right)}\left({\text{x}}_{0}\left(k\right)-{\text{x}}_{i}\left(k\right)\right). \end{array}$$

In this study, the control input of the *i*^th^ agent to ensure the consensus of leader-followers is proposed.

(5) $$\begin{array}{c} {\text{u}}_{i}\left(k\right)=\left({\text{K}}_{\sigma \left(k\right)}+{\text{K}}_{c,\sigma \left(k\right)}\right)\upsilon_{i}\left(k\right), \end{array}$$

where K_*σ*(*k*)_ is the control parameter to be determined by robust control theory, and K_*c*,*σ*(*k*)_ is the compensated parameter obtained by deep *Q* learning. In this study, the gained parameters K_*c*,*σ*(*k*)_ are supposed to vary in a finite set with given bounds. The K_*c*,*σ*(*k*)_ can be viewed as additional perturbance of K_*σ*(*k*)_, which can be described as follows:

(6) $$\begin{array}{c} {\text{K}}_{c,\sigma \left(k\right)}={\text{M}}_{\sigma \left(k\right)}{\text{F}}_{\sigma \left(k\right)}{\text{N}}_{\sigma \left(k\right)}, \end{array}$$

where M_*σ*(*k*)_ ∈ R^*l*×*l*_Δ_^ and N_*σ*(*k*)_ ∈ R^*q*_Δ_×*q*^ are the known matrices with appropriate dimensions, and F_*σ*(*k*)_ ∈ R^*l*_Δ_×*q*_Δ_^ are the unknown matrices with F_*σ*(*k*)_^T^F_*σ*(*k*)_ ≤ I.

For the *i*^th^ agent, the error of state is defined as e_*i*_(*k*)=x_*i*_(*k*) − x_0_(*k*). Then, the closed-loop system can be rewritten as

(7) $$\begin{array}{c} \left\{\begin{array}{c} \text{e}\left(k+1\right)={\tilde{\text{A}}}_{\sigma \left(k\right)}\text{e}\left(k\right)+\omega \tilde{B}\left(k\right),\\ \text{z}\left(k\right)={\tilde{\text{C}}}_{\sigma \left(k\right)}\text{e}\left(k\right), \end{array}\right) \end{array}$$

where e(*k*)=[e_1_^T^(*k*),…,e_*n*_^T^(*k*)]^T^, z(*k*)=[z_1_^T^(*k*),…,z_*n*_^T^(*k*)]^T^, *ω*(*k*)=[*ω*_1_^T^(*k*),…,*ω*_*n*_^T^(*k*)]^T^, ${\tilde{\text{A}}}_{\sigma \left(k\right)}={\text{I}}_{n}⊗\text{A}-\left({ℒ}_{\sigma \left(k\right)}+\Theta_{\sigma \left(k\right)}\right)⊗\text{B}\left({\text{K}}_{\sigma \left(k\right)}+{\text{K}}_{c,\sigma \left(k\right)}\right)$, $\tilde{B}={\text{I}}_{n}⊗\text{D}$, and ${\tilde{\text{C}}}_{\sigma \left(k\right)}=-\left({ℒ}_{\sigma \left(k\right)}+\Theta_{\sigma \left(k\right)}\right)⊗\text{C}$.

To facilitate the proof, the definitions and lemmas are given as follows.

**Definition 1 (see [37]).**

For given switching signal *σ*(*k*) and *k*_1_ > 0, define *N*_*σ*,*s*_(0, *k*_1_) as the number of switching instants over the time interval (0, *k*_1_). *T*_*σs*_(0, *k*_1_) is set to be the activated time of undirected graph *𝒢*_*s*_ during (0, *k*_1_). There exist constant scalars *N*_0_ ≥ 0 and *τ*_*as*_ > 0, such that

(8) $$\begin{array}{c} N_{\sigma ,s}\left(0,k_{1}\right)\leq N_{0}+\frac{T_{\sigma s}\left(0,k_{1}\right)}{\tau_{as}}. \end{array}$$

Then, *τ*_*as*_ is called the mode-dependent average dwell time and *N*_*σ*,*s*_(0, *k*_1_) is the mode-dependent chatter bound, respectively. In this study, we set *N*_0_=0.

**Definition 2 .**

(see [37]). If there exist control protocol in equation (5), all agents asymptotically track the state trajectory of the leader, such that

(9) $$\begin{array}{c} \underset{k⟶\infty }{\lim}{\left\|x_{i}\left(k\right)-x_{0}\left(k\right)\right\|}^{2}=0,\\ \text{for any initial conditions }x_{i}\left(0\right),i\in ℋ \end{array}$$

**Definition 3 .**

(see [38]). For given constant scalars 0 < *δ* < 1 and *γ* > 0, the prescribed *L*_2_ − *L*_*∞*_ attenuation performance *γ* is satisfied such that

1. The MASs in equations (2)-(3) are asymptotically stable when *ω*(*k*)=0.
2. The following inequation holds for all nonzero *ω*(*k*) ∈ *l*_2_(0, *∞*].

(10) $$\begin{array}{c} {\left\|z\left(r\right)\right\|}_{L_{\infty }}^{2}\leq \gamma^{2}{\left\|\omega \left(r\right)\right\|}_{L_{2}}^{2}. \end{array}$$

**Lemma 1 .**

(see [35]). The matrices **ℒ**_*σ*(*k*)_+Θ_*σ*(*k*)_ are symmetric and positive definite if and only if the graphs ${\tilde{𝒢}}_{\sigma \left(k\right)}$ are connected for *t* ≥ 0. Moreover, there exist a transformation matrix *T*_*σ*(*k*)_, such that the following equation holds.

(11) $$\begin{array}{c} {\text{T}}_{\sigma \left(k\right)}\left({ℒ}_{\sigma \left(k\right)}+\Theta_{\sigma \left(k\right)}\right){\text{T}}_{\sigma \left(k\right)}^{-1}={\text{Z}}_{\sigma \left(k\right)}=\text{diag}\left(\lambda_{1}^{\sigma \left(k\right)},\ldots ,\lambda_{n}^{\sigma \left(k\right)}\right), \end{array}$$

where *λ*_*i*_^*σ*(*k*)^, *i* ∈ ℋ are the nonzero eigenvalues of matrices **ℒ**_*σ*(*k*)_+Θ_*σ*(*k*)_.

**Lemma 2 .**

(see [39]). For given constant *a* > 0 and real matrices Θ,U,V,W, it is concluded that equation (12) is equivalent to equation (13).

(12) $$\begin{array}{c} \left[\begin{array}{cc} \Theta & \text{U}+a\text{V}\\ {}* & -a\left(\text{W}+{\text{W}}^{\text{T}}\right) \end{array}\right]<0, \end{array}$$

(13) $$\begin{array}{c} \Theta +\text{U}{\text{W}}^{-1}{\text{V}}^{\text{T}}+\text{V}{\text{W}}^{-\text{T}}{\text{U}}^{\text{T}}<0. \end{array}$$

**Lemma 3 (see [39]).**

For given symmetric matrix *𝒯* and matricesℳ, *𝒩*, if there exist constant scalar *ε* > 0, such that

(14) $$\begin{array}{c} T+ℳ{ℳ}^{T}\varepsilon^{-1}+\varepsilon N^{T}N<0 \end{array}$$

Then, the following equation holds for any appropriate ℱ with ℱ^*T*^ℱ ≤ *I*.

(15) $$\begin{array}{c} T+\mathrm{ℳℱ}N+N^{T}{ℱ}^{T}ℳ<0. \end{array}$$

## 3. Main Results

### 3.1. *L*_2_-*L*_∞_ Consensus Protocol Design

In this section, the *L*_2_-*L*_∞_ consensus protocol is proposed, and the stability and prescribed performance are guaranteed.

**Lemma 4 .**

For given constant scalars 0 < *δ* < 1, *γ* > 0. The system in (7) with control input in (5) is asymptotic stable with *L*_2_-*L*_∞_ attenuation performance *γ* if and only if the following equation holds.

(16) $$\begin{array}{c} \left\{\begin{array}{c} \tilde{\text{e}}\left(k+1\right)=\left({\text{I}}_{n}⊗\text{A}-{\text{Z}}_{\sigma \left(k\right)}⊗\text{B}\left({\text{K}}_{\sigma \left(k\right)}+\Delta {\text{K}}_{\sigma \left(k\right)}\right)\right)\tilde{\text{e}}\left(k\right)+\left({\text{I}}_{n}⊗\text{D}\right)\tilde{\omega }\left(k\right),\\ \tilde{\text{z}}\left(k\right)=-\left({\text{Z}}_{\sigma \left(k\right)}⊗\text{C}\right)\tilde{\text{e}}\left(k\right), \end{array}\right) \end{array}$$

where

(17) $$\begin{array}{c} \tilde{\omega }\left(k\right)=\left({\text{T}}_{\sigma \left(k\right)}⊗{\text{I}}_{l}\right)\omega \left(k\right),\\ \tilde{\text{z}}\left(k\right)=\left({\text{T}}_{\sigma \left(k\right)}⊗{\text{I}}_{q}\right)\text{z}\left(k\right),\\ \tilde{\text{e}}\left(k\right)=\left({\text{T}}_{\sigma \left(k\right)}⊗{\text{I}}_{p}\right)\text{e}\left(k\right). \end{array}$$

**Proof**

Substituting equation (17) to (7), one can obtain equation (16). It can be inferred that the transformation matrix T_*σ*(*k*)_ is unique; therefore, we have the following equations.

(18) $$\begin{array}{c} {\left\|\tilde{\omega }\left(k\right)\right\|}_{L_{2}}^{2}=\sum_{0}^{\infty }{\left\|\tilde{\omega }\left(k\right)\right\|}_{2}^{2},\\ =\sum_{0}^{\infty }\omega^{\text{T}}\left(k\right){\left({\text{T}}_{\sigma \left(k\right)}⊗{\text{I}}_{l}\right)}^{\text{T}}\left({\text{T}}_{\sigma \left(k\right)}⊗{\text{I}}_{l}\right)\omega \left(k\right),\\ ={\left\|\omega \left(k\right)\right\|}_{L_{2}}^{2}, \end{array}$$

(19) $$\begin{array}{c} {\left\|\tilde{\text{z}}\left(r\right)\right\|}_{L_{\infty }}^{2}=\text{sup}\left\{{\tilde{\text{z}}}^{\text{T}}\left(k\right)\tilde{\text{z}}\left(k\right)\right\},\\ =\sup\left\{{\text{z}}^{\text{T}}\left(k\right){\left({\text{T}}_{\sigma \left(k\right)}⊗{\text{I}}_{l}\right)}^{\text{T}}\left({\text{T}}_{\sigma \left(k\right)}⊗{\text{I}}_{l}\right)\text{z}\left(k\right)\right\},\\ ={\left\|\text{z}\left(r\right)\right\|}_{L_{\infty }}^{2}. \end{array}$$

It is obvious that the problem of robust consensus protocol design can be converted to the controller design of (16).

**Remark 1 .**

The system in equation (16) consists of the independent system in equation (20). Therefore, the stability of equation (7) is equivalent to the stability of *n* subsystems in equation (20); the attempt to ensure the prescribed attenuation performance of (7) can be converted to guarantee the attenuation performance of (16).

(20) $$\begin{array}{c} \left\{\begin{array}{c} {\tilde{\text{e}}}_{i}\left(k+1\right)={\bar{\text{A}}}_{i}{\tilde{\text{e}}}_{i}\left(k\right)+\text{D}{\tilde{\omega }}_{i}\left(k\right)\\ {\tilde{\text{z}}}_{i}\left(k\right)={\bar{\text{C}}}_{i}{\tilde{\text{e}}}_{i}\left(k\right) \end{array},\;i\in ℋ\right), \end{array}$$

where ${\bar{\text{A}}}_{i}=\left(\text{A}-\lambda_{i}^{\sigma \left(k\right)}\text{B}\left({\text{K}}_{\sigma \left(k\right)}+{\text{K}}_{c,\sigma \left(k\right)}\right)\right)$, and ${\bar{\text{C}}}_{i}=-\lambda_{i}^{\sigma \left(k\right)}\text{C}$.

In Theorem 1, the sufficient conditions to guarantee the stability and prescribed attenuation performance index are presented.

**Theorem 1 .**

For given constant scalars *μ*_*s*_ > 1, 0 < *δ*_*s*_ < 1, *γ* > 0, if there exist Lyapunov functions $V_{i,s}\left({\tilde{\text{e}}}_{i}\left(k\right)\right),\;i\in ℋ$, and class functions *κ*_1_, *κ*_2_, the switched systems in equation (20) with MDADT satisfying equation (25) are globally uniformly asymptotically stable with prescribed *L*_2_-*L*_∞_ attenuation performance *γ*, such that

(21) $$\begin{array}{c} \kappa_{1}\left(\left\|{\tilde{\text{e}}}_{i}\left(k\right)\right\|\right)\leq V_{i,s}\left({\tilde{\text{e}}}_{i}\left(k\right)\right)\leq \kappa_{2}\left(\left\|{\tilde{\text{e}}}_{i}\left(k\right)\right\|\right), \end{array}$$

(22) $$\begin{array}{c} V_{i,s}\left({\tilde{\text{e}}}_{i}\left(k\right)\right)\leq \mu_{s}V_{i,r}\left({\tilde{\text{e}}}_{i}\left(k\right)\right),\;s\neq r, \end{array}$$

(23) $$\begin{array}{c} \Delta V_{i,s}\left({\tilde{\text{e}}}_{i}\left(k\right)\right)\leq -\delta_{s}V_{i,s}\left({\tilde{\text{e}}}_{i}\left(k\right)\right)+{\left\|\tilde{\omega }\left(k\right)\right\|}_{2}^{2}, \end{array}$$

(24) $$\begin{array}{c} {\left\|{\tilde{\text{z}}}_{i}\left(k\right)\right\|}_{2}^{2}\leq \gamma^{2}V_{i,s}\left({\tilde{\text{e}}}_{i}\left(k\right)\right), \end{array}$$

(25) $$\begin{array}{c} \tau_{as}\geq \tau_{as}^{*}=-\frac{\ln\;\mu_{s}}{\ln\left(1-\delta_{s}\right)}. \end{array}$$

**Proof**

The entire proof can be divided into two steps.

(1) The stability of equation (20).
The switching instants in the time interval (0, *k*) are set to be *k*_1_, *k*_2_,…, *k*_*t*_ with *k*_*t*+1_=*k*. Then, (26) holds when ‖*ω*_*i*_(*k*)‖ ≡ 0.
  (26) $$\begin{array}{c} V_{i,\sigma \left(k_{t}\right)}\left({\tilde{\text{e}}}_{i}\left(k\right)\right)\leq {\left(1-\delta_{\sigma \left(k_{t}\right)}\right)}^{k-k_{t}}V_{i,\sigma \left(k_{t}\right)}\left({\tilde{\text{e}}}_{i}\left(k_{t}\right)\right). \end{array}$$
Together with (22), we can conclude that
  (27) $$\begin{array}{c} V_{i,\sigma \left(k_{t}\right)}\left({\tilde{\text{e}}}_{i}\left(k\right)\right)\leq \mu_{\sigma \left(k_{t}\right)}{\left(1-\delta_{\sigma \left(k_{t}\right)}\right)}^{k-k_{t}}V_{i,\sigma \left(k_{t}\right)}\left({\tilde{\text{e}}}_{i}\left(k_{t}\right)\right). \end{array}$$
Based on equations (26)-(27), the following equation can be obtained by iteration.
  (28) $$\begin{array}{c} V_{i,\sigma \left(k_{t}\right)}\left({\tilde{\text{e}}}_{i}\left(k\right)\right)\leq \mu_{\sigma \left(k_{t}\right)}{\left(1-\delta_{\sigma \left(k_{t}\right)}\right)}^{k-k_{t}}V_{i,\sigma \left(k_{t}\right)}\left({\tilde{\text{e}}}_{i}\left(k_{t}\right)\right),\\ \leq \mu_{\sigma \left(k_{t}\right)}\mu_{\sigma \left(k_{t-1}\right)}{\left(1-\delta_{\sigma \left(k_{t}\right)}\right)}^{k-k_{t}}{\left(1-\delta_{\sigma \left(k_{t-1}\right)}\right)}^{k_{t}-k_{t-1}}V_{i,\sigma \left(k_{t-1}\right)}\left({\tilde{\text{e}}}_{i}\left(k_{t-1}\right)\right),\\ \cdot \cdot \cdot \\ \leq \prod_{r=0}^{t}\left\{\mu_{\sigma \left(k_{r}\right)}{\left(1-\delta_{\sigma \left(k_{r}\right)}\right)}^{k_{r+1}-k_{r}}\right\}V_{i,\sigma \left(0\right)}\left({\tilde{\text{e}}}_{i}\left(0\right)\right),\\ \leq \prod_{s=1}^{N_{f}}\left\{\mu_{\sigma \left(k_{s}\right)}^{N_{\sigma ,s}\left(0,k\right)}{\left(1-\delta_{\sigma \left(k_{s}\right)}\right)}^{T_{\sigma s}\left(0,k\right)}\right\}V_{i,\sigma \left(0\right)}\left({\tilde{\text{e}}}_{i}\left(0\right)\right). \end{array}$$
Combining with Definition 1, we have
  (29) $$\begin{array}{c} V_{i,\sigma \left(k_{t}\right)}\left({\tilde{\text{e}}}_{i}\left(k\right)\right)\leq \;\exp\left\{\overset{N_{f}}{\underset{s=1}{\sum }}\left(N_{\sigma ,s}\left(0,k\right)\ln\;\mu_{\sigma \left(k_{s}\right)}+T_{\sigma s}\left(0,k\right)\ln\left(1-\delta_{\sigma \left(k_{s}\right)}\right)\right)\right\}V_{i,\sigma \left(0\right)}\left({\tilde{\text{e}}}_{i}\left(0\right)\right),\\ \leq \;\exp\left\{\overset{N_{f}}{\underset{s=1}{\sum }}\left(\frac{T_{\sigma s}\left(0,k\right)}{\tau_{as}}\ln\;\mu_{\sigma \left(k_{s}\right)}+T_{\sigma s}\left(0,k\right)\ln\left(1-\delta_{\sigma \left(k_{s}\right)}\right)\right)\right\}V_{i,\sigma \left(0\right)}\left({\tilde{\text{e}}}_{i}\left(0\right)\right),\\ \leq \;\exp\left\{\overset{N_{f}}{\underset{s=1}{\sum }}\left(T_{\sigma s}\left(0,k\right)\left(\frac{\ln\;\mu_{\sigma \left(k_{s}\right)}}{\tau_{as}}+\ln\left(1-\delta_{\sigma \left(k_{s}\right)}\right)\right)\right)\right\}V_{i,\sigma \left(0\right)}\left({\tilde{\text{e}}}_{i}\left(0\right)\right). \end{array}$$
Then, we can obtain (29) based on (21).
  (30) $$\begin{array}{c} {\left\|{\tilde{\text{e}}}_{i}\left(k\right)\right\|}^{2}\leq \kappa_{1}^{-1}\kappa_{2}\exp\left\{\sum_{s=1}^{N_{f}}\left(T_{\sigma s}\left(0,k\right)\left(\frac{\ln\;\mu_{\sigma \left(k_{s}\right)}}{\tau_{as}}+\ln\left(1-\delta_{\sigma \left(k_{s}\right)}\right)\right)\right)\right\}{\left\|{\tilde{\text{e}}}_{i}\left(0\right)\right\|}^{2}. \end{array}$$
Therefore, the system in (20) with MDADT satisfying (25) is globally uniformly asymptotically stable.
(2) The system in equation (20) has prescribed *L*_2_-*L*_∞_ attenuation performance *γ*.

Together with equations (22)-(23), one has

(31) $$\begin{array}{c} V_{i,\sigma \left(k_{t}\right)}\left({\tilde{\text{e}}}_{i}\left(k\right)\right)\leq {\left(1-\delta_{\sigma \left(k_{t}\right)}\right)}^{k-k_{t}}V_{i,\sigma \left(k_{t}\right)}\left({\tilde{\text{e}}}_{i}\left(k_{t}\right)\right)+\overset{k-1}{\underset{r=k_{t}}{\sum }}{\left(1-\delta_{\sigma \left(k_{t}\right)}\right)}^{k-r-1}{\left\|\tilde{\omega }\left(r\right)\right\|}_{2}^{2},\\ \leq \mu_{\sigma \left(k_{t}\right)}{\left(1-\delta_{\sigma \left(k_{t}\right)}\right)}^{k-k_{t}}V_{i,\sigma \left(k_{t-1}\right)}\left({\tilde{\text{e}}}_{i}\left(k_{t}\right)\right)+\overset{k-1}{\underset{r=k_{t}}{\sum }}{\left(1-\delta_{\sigma \left(k_{t}\right)}\right)}^{k-r-1}{\left\|\tilde{\omega }\left(r\right)\right\|}_{2}^{2}. \end{array}$$

Then, one can obtain the equation as follows by iteration.

(32) $$\begin{array}{c} V_{i,\sigma \left(k_{t}\right)}\left({\tilde{\text{e}}}_{i}\left(k\right)\right)\leq \mu_{\sigma \left(k_{t}\right)}{\left(1-\delta_{\sigma \left(k_{t}\right)}\right)}^{k-k_{t}}V_{i,\sigma \left(k_{t-1}\right)}\left({\tilde{\text{e}}}_{i}\left(k_{t}\right)\right)+\overset{k-1}{\underset{r=k_{t}}{\sum }}{\left(1-\delta_{\sigma \left(k_{t}\right)}\right)}^{k-r-1}{\left\|\tilde{\omega }\left(r\right)\right\|}_{2}^{2},\\ \leq \mu_{\sigma \left(k_{t}\right)}{\left(1-\delta_{\sigma \left(k_{t}\right)}\right)}^{k-k_{t}}\left[\mu_{\sigma \left(k_{t-1}\right)}{\left(1-\delta_{\sigma \left(k_{t-1}\right)}\right)}^{k_{t}-k_{t-1}}V_{i,\sigma \left(k_{t-2}\right)}\left({\tilde{\text{e}}}_{i}\left(k_{t-1}\right)\right),\right)\\ \left(+\overset{k_{t}-1}{\underset{r=k_{t-1}}{\sum }}{\left(1-\delta_{\sigma \left(k_{t-1}\right)}\right)}^{k-r-1}{\left\|\tilde{\omega }\left(r\right)\right\|}_{2}^{2}\right]+\overset{k-1}{\underset{r=k_{t}}{\sum }}{\left(1-\delta_{\sigma \left(k_{t}\right)}\right)}^{k-r-1}{\left\|\tilde{\omega }\left(r\right)\right\|}_{2}^{2},\\ \cdot \cdot \cdot \\ \leq \mu_{\sigma \left(k_{1}\right)}\cdot \cdot \cdot \mu_{\sigma \left(k_{t}\right)}{\left(1-\delta_{\sigma \left(k_{0}\right)}\right)}^{k_{1}-k_{0}}\cdot \cdot \cdot {\left(1-\delta_{\sigma \left(k_{t}\right)}\right)}^{k-k_{t}}V_{i,\sigma \left(0\right)}\left({\tilde{\text{e}}}_{i}\left(0\right)\right),\\ +\mu_{\sigma \left(k_{1}\right)}\cdot \cdot \cdot \mu_{\sigma \left(k_{t}\right)}{\left(1-\delta_{\sigma \left(k_{0}\right)}\right)}^{k_{1}-k_{0}}\cdot \cdot \cdot {\left(1-\delta_{\sigma \left(k_{t}\right)}\right)}^{k-k_{t}}\overset{k_{1}-1}{\underset{r=k_{0}}{\sum }}{\left(1-\delta_{\sigma \left(k_{0}\right)}\right)}^{k_{1}-r-1}{\left\|\tilde{\omega }\left(r\right)\right\|}_{2}^{2},\\ +\mu_{\sigma \left(k_{2}\right)}\cdot \cdot \cdot \mu_{\sigma \left(k_{t}\right)}{\left(1-\delta_{\sigma \left(k_{1}\right)}\right)}^{k_{2}-k_{1}}\cdot \cdot \cdot {\left(1-\delta_{\sigma \left(k_{t}\right)}\right)}^{k-k_{t}}\overset{k_{2}-1}{\underset{r=k_{1}}{\sum }}{\left(1-\delta_{\sigma \left(k_{1}\right)}\right)}^{k_{2}-r-1}{\left\|\tilde{\omega }\left(r\right)\right\|}_{2}^{2},\\ +\cdot \cdot \cdot +\overset{k-1}{\underset{r=k_{t}}{\sum }}{\left(1-\delta_{\sigma \left(k_{t}\right)}\right)}^{k-r-1}{\left\|\tilde{\omega }\left(r\right)\right\|}_{2}^{2},\\ \leq {\left(1-\delta_{\sigma \left(k_{t}\right)}\right)}^{k-k_{t}}\prod_{r=0}^{t-1}\left\{\mu_{\sigma \left(k_{r+1}\right)}{\left(1-\delta_{\sigma \left(k_{r}\right)}\right)}^{k_{r+1}-k_{r}}\right\}V_{i,\sigma \left(0\right)}\left({\tilde{\text{e}}}_{i}\left(0\right)\right),\\ +\mu_{\sigma \left(k_{t}\right)}{\left(1-\delta_{\sigma \left(k_{t}\right)}\right)}^{k-k_{t}}\overset{t-1}{\underset{q=1}{\sum }}\left(\prod_{p=q}^{t-1}\mu_{\sigma \left(k_{p}\right)}{\left(1-\delta_{\sigma \left(k_{p}\right)}\right)}^{k_{p+1}-k_{p}}\overset{k_{q}-1}{\underset{r=k_{q-1}}{\sum }}{\left(1-\delta_{\sigma \left(k_{q-1}\right)}\right)}^{k_{q}-r-1}{\left\|\tilde{\omega }\left(r\right)\right\|}_{2}^{2}\right),\\ +\mu_{\sigma \left(k_{t}\right)}{\left(1-\delta_{\sigma \left(k_{t}\right)}\right)}^{k-k_{t}}\overset{k_{t}-1}{\underset{r=k_{t-1}}{\sum }}{\left(1-\delta_{\sigma \left(k_{t-1}\right)}\right)}^{k_{t}-r-1}{\left\|\tilde{\omega }\left(r\right)\right\|}_{2}^{2},\\ +\overset{k-1}{\underset{r=k_{t}}{\sum }}{\left(1-\delta_{\sigma \left(k_{t}\right)}\right)}^{k-r-1}{\left\|\tilde{\omega }\left(r\right)\right\|}_{2}^{2},\\ \leq \prod_{s=1}^{N_{f}}\left\{\mu_{\sigma \left(k_{s}\right)}^{N_{\sigma ,s}\left(0,k\right)}{\left(1-\delta_{\sigma \left(k_{s}\right)}\right)}^{T_{\sigma s}\left(0,k\right)}\right\}V_{i,\sigma \left(0\right)}\left({\tilde{\text{e}}}_{i}\left(0\right)\right),\\ +\sum_{q=1}^{t}\left(\prod_{s=1}^{N_{f}}\left\{\mu_{\sigma \left(k_{s}\right)}^{N_{\sigma ,s}\left(k_{q},k\right)}{\left(1-\delta_{\sigma \left(k_{s}\right)}\right)}^{T_{\sigma s}\left(k_{q},k\right)}\right\}\sum_{r=k_{q-1}}^{k_{q}-1}{\left(1-\delta_{\sigma \left(k_{q-1}\right)}\right)}^{k_{q}-r-1}{\left\|\tilde{\omega }\left(r\right)\right\|}_{2}^{2}\right),\\ +\sum_{r=k_{t}}^{k-1}{\left(1-\delta_{\sigma \left(k_{t}\right)}\right)}^{k-r-1}{\left\|\tilde{\omega }\left(r\right)\right\|}_{2}^{2}. \end{array}$$

Substituting the equation above into (8), one can obtain that

(33) $$\begin{array}{c} V_{i,\sigma \left(k_{t}\right)}\left({\tilde{\text{e}}}_{i}\left(k\right)\right)\leq \;\exp\left\{\overset{N_{f}}{\underset{s=1}{\sum }}T_{\sigma s}\left(0,k\right)\left(\frac{\ln\;\mu_{\sigma \left(k_{s}\right)}}{\tau_{as}}+\ln\left(1-\delta_{\sigma \left(k_{s}\right)}\right)\right)\right\}V_{i,\sigma \left(0\right)}\left({\tilde{\text{e}}}_{i}\left(0\right)\right),\\ +\sum_{q=1}^{t}\left\{\exp\left(\overset{N_{f}}{\underset{s=1}{\sum }}T_{\sigma s}\left(k_{q},k\right)\left(\frac{\ln\;\mu_{\sigma \left(k_{s}\right)}}{\tau_{as}}+\ln\left(1-\delta_{\sigma \left(k_{s}\right)}\right)\right)\right)\right),\\ \times \left(\overset{k_{q}-1}{\underset{r=k_{q-1}}{\sum }}{\left(1-\delta_{\sigma \left(k_{q-1}\right)}\right)}^{k_{q}-r-1}{\left\|\tilde{\omega }\left(r\right)\right\|}_{2}^{2}\right\},\\ +\overset{k-1}{\underset{r=k_{t}}{\sum }}{\left(1-\delta_{\sigma \left(k_{t}\right)}\right)}^{k-r-1}{\left\|\tilde{\omega }\left(r\right)\right\|}_{2}^{2}. \end{array}$$

According to the conditions *μ*_*s*_ > 1, 0 < *δ*_*s*_ < 1, and (25), we have

(34) $$\begin{array}{c} 0<1-\delta_{\sigma \left(k_{s}\right)}<1, \end{array}$$

(35) $$\begin{array}{c} 0<\;\exp\left(\sum_{s=1}^{N_{f}}T_{\sigma s}\left(k_{q},k\right)\left(\frac{\ln\;\mu_{\sigma \left(k_{s}\right)}}{\tau_{as}}+\ln\left(1-\delta_{\sigma \left(k_{s}\right)}\right)\right)\right)<1. \end{array}$$

Combining equations (32)–(34), one can obtain equation (35).

(36) $$\begin{array}{c} V_{i,\sigma \left(k_{t}\right)}\left({\tilde{\text{e}}}_{i}\left(k\right)\right)\leq \sum_{q=1}^{t}\sum_{r=k_{q-1}}^{k_{q}-1}{\left\|\tilde{\omega }\left(r\right)\right\|}_{2}^{2}+\sum_{r=k_{t}}^{k-1}{\left\|\tilde{\omega }\left(r\right)\right\|}_{2}^{2},\\ =\sum_{r=0}^{k_{t}-1}{\left\|\tilde{\omega }\left(r\right)\right\|}_{2}^{2}+\sum_{r=k_{t}}^{k-1}{\left\|\tilde{\omega }\left(r\right)\right\|}_{2}^{2},\\ =\sum_{r=0}^{k-1}{\left\|\tilde{\omega }\left(r\right)\right\|}_{2}^{2}. \end{array}$$

Together with (24), it is obvious that

(37) $$\begin{array}{c} {\left\|{\tilde{\text{z}}}_{i}\left(k\right)\right\|}_{2}^{2}\leq \gamma^{2}V_{i,s}\left({\tilde{\text{e}}}_{i}\left(k\right)\right),\\ \leq \gamma^{2}\sum_{r=0}^{k-1}{\left\|\tilde{\omega }\left(r\right)\right\|}_{2}^{2}, \end{array}$$

which implies that $\sup\left\{{\left\|\tilde{\text{z}}\left(r\right)\right\|}_{2}^{2}\right\}\leq \gamma^{2}{\left\|\tilde{\omega }\left(r\right)\right\|}_{L_{2}}^{2}$, and the proof is complete.

**Corollary 1 .**

For given constant scalars *μ*_*s*_ > 1, 0 < *δ*_*s*_ < 1, *γ* > 0, if there exist positive-definite matrices P_*i*,*s*_ ∈ R^*p*×*p*^ satisfying equations (37)–(39), the switched systems in equation (20) with MDADT satisfying equation (25) are globally uniformly asymptotically stable with prescribed *L*_2_-*L*_∞_ attenuation performance *γ*.

(38) $$\begin{array}{c} {\text{P}}_{i,s}\leq \mu_{s}{\text{P}}_{i,r},\;s\neq r, \end{array}$$

(39) $$\begin{array}{c} \left[\begin{array}{cc} {\bar{\text{A}}}_{i}^{\text{T}}{\text{P}}_{i,s}{\bar{\text{A}}}_{i}-\left(1-\delta_{s}\right){\text{P}}_{i,s} & {\bar{\text{A}}}_{i}^{\text{T}}{\text{P}}_{i,s}\text{D}\\ {}* & {\text{D}}^{\text{T}}{\text{P}}_{i,s}\text{D}-\text{I} \end{array}\right]\leq 0, \end{array}$$

(40) $$\begin{array}{c} {\left(\lambda_{i}^{s}\right)}^{2}{\text{C}}^{\text{T}}\text{C}<\gamma^{2}{\text{P}}_{i,s}. \end{array}$$

**Proof**

The Lyapunov function $V_{i,s}\left({\tilde{\text{e}}}_{i}\left(k\right)\right),\;i\in ℋ$, is defined as follows:

(41) $$\begin{array}{c} V_{i,s}\left({\tilde{\text{e}}}_{i}\left(k\right)\right)={\tilde{\text{e}}}_{i}^{\text{T}}\left(k\right){\text{P}}_{i,s}{\tilde{\text{e}}}_{i}\left(k\right). \end{array}$$

According to (20) and (39), we can conclude that (38) is equivalent to (24). Along the trajectory of $V_{i,s}\left({\tilde{\text{e}}}_{i}\left(k\right)\right)$, one has

(42) $$\begin{array}{c} \Delta V_{i,s}\left({\tilde{\text{e}}}_{i}\left(k\right)\right)={\tilde{\text{e}}}_{i}^{\text{T}}\left(k+1\right){\text{P}}_{i,s}{\tilde{\text{e}}}_{i}\left(k+1\right)-{\tilde{\text{e}}}_{i}^{\text{T}}\left(k\right){\text{P}}_{i,s}{\tilde{\text{e}}}_{i}\left(k\right),\\ ={\left({\bar{\text{A}}}_{i}{\tilde{\text{e}}}_{i}\left(k\right)+\text{D}{\tilde{\omega }}_{i}\left(k\right)\right)}^{\text{T}}\times {\text{P}}_{i,s}\left({\bar{\text{A}}}_{i}{\tilde{\text{e}}}_{i}\left(k\right)+\text{D}{\tilde{\omega }}_{i}\left(k\right)\right),\\ -{\tilde{\text{e}}}_{i}^{\text{T}}\left(k\right){\text{P}}_{i,s}{\tilde{\text{e}}}_{i}\left(k\right),\\ ={\tilde{\text{e}}}_{i}^{\text{T}}\left(k\right)\left[{\bar{\text{A}}}_{i}^{\text{T}}{\text{P}}_{i,s}{\bar{\text{A}}}_{i}-{\text{P}}_{i,s}\right]{\tilde{\text{e}}}_{i}\left(k\right)+{\tilde{\omega }}_{i}^{\text{T}}\left(k\right)\left[{\text{D}}^{\text{T}}{\text{P}}_{i,s}\text{D}\right]{\tilde{\omega }}_{i}\left(k\right),\\ +{\tilde{\text{e}}}_{i}^{\text{T}}\left(k\right){\bar{\text{A}}}_{i}^{\text{T}}{\text{P}}_{i,s}\text{D}{\tilde{\omega }}_{i}\left(k\right)+{\tilde{\omega }}_{i}^{\text{T}}\left(k\right){\text{D}}^{\text{T}}{\text{P}}_{i,s}{\bar{\text{A}}}_{i}{\tilde{\text{e}}}_{i}\left(k\right). \end{array}$$

Together with equations (40)-(41), we have

(43) $$\begin{array}{c} \Delta V_{i,s}\left({\tilde{\text{e}}}_{i}\left(k\right)\right)+\delta_{s}V_{i,s}\left({\tilde{\text{e}}}_{i}\left(k\right)\right)-{\tilde{\omega }}_{i}^{\text{T}}\left(k\right){\tilde{\omega }}_{i}\left(k\right),\\ ={\tilde{\text{e}}}_{i}^{\text{T}}\left(k\right)\left[{\bar{\text{A}}}_{i}^{\text{T}}{\text{P}}_{i,s}{\bar{\text{A}}}_{i}-\left(1-\delta_{s}\right){\text{P}}_{i,s}\right]{\tilde{\text{e}}}_{i}\left(k\right)+{\tilde{\text{e}}}_{i}^{\text{T}}\left(k\right){\bar{\text{A}}}_{i}^{\text{T}}{\text{P}}_{i,s}\text{D}{\tilde{\omega }}_{i}\left(k\right),\\ +{\tilde{\omega }}_{i}^{\text{T}}\left(k\right){\text{D}}^{\text{T}}{\text{P}}_{i,s}{\bar{\text{A}}}_{i}{\tilde{\text{e}}}_{i}\left(k\right)+{\tilde{\omega }}_{i}^{\text{T}}\left(k\right)\left[{\text{D}}^{\text{T}}{\text{P}}_{i,s}{\text{D}}_{i}-\text{I}\right]{\tilde{\omega }}_{i}\left(k\right),\\ ={\left[\begin{array}{c} {\tilde{\text{e}}}_{i}\left(k\right)\\ {\tilde{\omega }}_{i}\left(k\right) \end{array}\right]}^{\text{T}}\left[\begin{array}{cc} {\bar{\text{A}}}_{i}^{\text{T}}{\text{P}}_{i,s}{\bar{\text{A}}}_{i}-\left(1-\delta_{s}\right){\text{P}}_{i,s} & {\bar{\text{A}}}_{i}^{\text{T}}{\text{P}}_{i,s}\text{D}\\ {}* & {\text{D}}^{\text{T}}{\text{P}}_{i,s}\text{D}-\text{I} \end{array}\right]\left[\begin{array}{c} {\tilde{\text{e}}}_{i}\left(k\right)\\ {\tilde{\omega }}_{i}\left(k\right) \end{array}\right],\\ \leq 0. \end{array}$$

According to Theorem 1, we can conclude that the system in (20) with MDADT satisfying (25) is globally uniformly asymptotically stable with prescribed *L*_2_-*L*_∞_ attenuation performance *γ*.

Based on Theorem 1 and Corollary 1, the solutions of consensus protocol are given in Theorem 2.

**Theorem 2 .**

For given constant scalars *μ*_*s*_ > 1, 0 < *δ*_*s*_ < 1, *γ* > 0, *a*_*s*_ > 0, and *ε*_*s*_ > 0, if there exist positive-definite matrices P_*i*,*s*_ ∈ R^*p*×*p*^, matrices X_*s*_ ∈ R^*l*×*l*^, Y_*s*_ ∈ R^*l*×*q*^, the MASs in (2)-(3) with control input in equation (5) are asymptotically stable with prescribed *L*_2_-*L*_∞_ attenuation performance *γ* such that equation (43) holds.

(44) $$\begin{array}{c} \left[\begin{array}{ccccc} \Xi_{11} & 0 & \Xi_{13} & a_{s}\lambda_{i}^{s}{\text{Y}}_{s}^{\text{T}} & 0\\ {}* & -\text{I} & {\text{D}}^{\text{T}}{\text{P}}_{i,s} & 0 & 0\\ {}* & * & -\left(I_{n-1}⊗P_{s}\right) & \text{B}{\text{X}}_{s}-{\text{P}}_{i,s}\text{B} & {\text{P}}_{i,s}\text{B}{\text{M}}_{s}\\ {}* & * & * & -a_{s}\left({\text{X}}_{s}+{\text{X}}_{s}^{\text{T}}\right) & 0\\ {}* & * & * & * & -\varepsilon_{s}\text{I} \end{array}\right]<0. \end{array}$$

The parameters of control protocol can be derived in (43).

(45) $$\begin{array}{c} {\text{K}}_{s}={\text{X}}_{s}^{-1}{\text{Y}}_{s}, \end{array}$$

where *Ξ*_11_=−(1 − *δ*_*s*_)P_*i*,*s*_+*ε*_*s*_(*λ*_*i*_^*s*^)^2^N_*s*_^T^N_*s*_, *Ξ*_13_=A^T^P_*i*,*s*_ − *λ*_*i*_^*s*^Y_*s*_^T^B^T^.

**Proof**

According to Schur complement, it is obvious that equation (43) is equivalent to equation (45).

(46) $$\begin{array}{c} \left[\begin{array}{cccc} \Xi_{11} & 0 & \Xi_{13} & a_{s}\lambda_{i}^{s}{\text{Y}}_{s}^{\text{T}}\\ {}* & -\text{I} & {\text{D}}^{\text{T}}{\text{P}}_{i,s} & 0\\ {}* & * & -{\text{P}}_{i,s} & \text{B}{\text{X}}_{s}-{\text{P}}_{i,s}\text{B}\\ {}* & * & * & -a_{s}\left({\text{X}}_{s}+{\text{X}}_{s}^{\text{T}}\right) \end{array}\right]+\varepsilon_{s}^{-1}\left[\begin{array}{c} 0\\ 0\\ {\text{P}}_{i,s}\text{B}{\text{M}}_{s}\\ 0 \end{array}\right]{\left[\begin{array}{c} 0\\ 0\\ {\text{P}}_{i,s}\text{B}{\text{M}}_{s}\\ 0 \end{array}\right]}^{\text{T}},\\ =\left[\begin{array}{cccc} \Xi_{11} & 0 & \Xi_{13} & a_{s}\lambda_{i}^{s}{\text{Y}}_{s}^{\text{T}}\\ {}* & -\text{I} & {\text{D}}^{\text{T}}{\text{P}}_{i,s} & 0\\ {}* & * & -{\text{P}}_{i,s}+\varepsilon_{s}^{-1}{\text{P}}_{i,s}\text{B}{\text{M}}_{s}{\text{M}}_{s}^{\text{T}}{\text{B}}^{\text{T}}{\text{P}}_{i,s} & \text{B}{\text{X}}_{s}-{\text{P}}_{i,s}\text{B}\\ {}* & * & * & -a_{s}\left({\text{X}}_{s}+{\text{X}}_{s}^{\text{T}}\right) \end{array}\right],\\ <0. \end{array}$$

Define Θ_*s*_=*𝒯*_*s*_+*ε*_*s*_^−1^ℳ_*s*_^*T*^+*ε*_*s*_*𝒩*_*s*_^*T*^*𝒩*_*s*_, ${\text{U}}_{s}={\left[\begin{array}{ccc} 0 & 0 & {\text{X}}_{s}^{\text{T}}{\text{B}}^{\text{T}}-{\text{B}}^{\text{T}}{\text{P}}_{i,s} \end{array}\right]}^{\text{T}}$, and $V_{s}={\left[\begin{array}{ccc} \lambda_{i}^{s}Y_{s} & 0 & 0 \end{array}\right]}^{T}$, where ${𝒯}_{s}=\left[\begin{array}{ccc} -\left(1-\delta_{s}\right)P_{i,s} & 0 & A^{T}P_{i,s}-\lambda_{i}^{s}Y_{s}^{t}B^{T}\\ {}* & -I & D^{T}P_{i,s}\\ {}* & * & -P_{i,s} \end{array}\right]$, ${ℳ}_{s}=\left[\begin{array}{c} 0\\ 0\\ P_{i,s}BM_{s} \end{array}\right]$, and ${𝒩}_{s}=\left[\begin{array}{ccc} -\lambda_{i}^{s}N_{s} & 0 & 0 \end{array}\right]$.

Together with Lemma 2, we have

(47) $$\begin{array}{c} \Theta_{s}+U_{s}X_{s}^{-1}V_{s}^{T}+V_{s}X_{s}^{-T}U_{s}^{T},\\ =T_{s}+\varepsilon_{s}^{-1}{ℳ}_{s}{ℳ}_{s}^{T}+\varepsilon_{s}N_{s}^{T}N_{s}+U_{s}X_{s}^{-1}V_{s}^{T}+V_{s}X_{s}^{-T}U_{s}^{T}<0. \end{array}$$

Moreover, based on Lemma 3, one has

(48) $$\begin{array}{c} T+\mathrm{ℳℱ}N+N^{T}{ℱ}^{T}{ℳ}^{T}+U_{s}X_{s}^{-1}V_{s}^{T}+V_{s}X_{s}^{-T}U_{s}^{T}\\ =\left[\begin{array}{ccc} -\left(1-\delta_{s}\right)P_{i,s} & 0 & {\bar{A}}_{i}^{T}P_{i,s}\\ {}* & -I & D^{T}P_{i,s}\\ {}* & * & -P_{i,s} \end{array}\right]<0. \end{array}$$

According to Schur complement, it is obvious that (46) is equivalent to (37), which completes the proof.

## 4. Compensated Consensus Protocol Design Based on FDQL

In this section, the learning-based consensus protocol is proposed based on deep reinforcement learning, where fuzzy deep *Q* learning is utilized. The stability and prescribed attenuation performance are guaranteed by the robust control theory, and the learning-based control protocol is introduced to improve the transient performance and realize optimal control policy. The output of the learning-based control protocol can be viewed as an additional variation of robust consensus protocol. The online scheduling of control protocol is established as a Markovian process. Therefore, the advantages of robust control theory and deep reinforcement learning are combined.

It is well known that reinforcement learning is composed of state, action, agent, and environment. The state of *k*^th^ step is defined as *s*_*k*_, and the chosen action is supposed to be *a*_*k*_; then, the reward function *r*_*k*_ and the state *s*_*k*+1_ are generated based on the interaction with the environment. Therefore, the optimal control policy can be obtained by maximum the reward function.

To improve the convergence of consensus protocol, the state is defined as $s_{k}=\left[\begin{array}{cc} {\tilde{\text{e}}}_{i}\left(k\right) & {\tilde{\text{z}}}_{i}\left(k\right) \end{array}\right]$ and the action is defined as *a*_*k*_=[K_*c*,*σ*(*k*)_].

In *Q* learning, the deep neural network is utilized to approximate the action-state value function *Q*^*∗*^(*s*_*k*_, *a*_*k*_), which can be described as

(49) $$\begin{array}{c} f\left(s_{k},a_{k},\omega \right)=Q^{*}\left(s_{k},a_{k}\right), \end{array}$$

where *f*(*s*_*k*_, *a*_*k*_, *ω*) denotes the function of deep neural networks.

The action is chosen based on the maximum *Q* value:

(50) $$\begin{array}{c} a^{*}=\arg\;\underset{a}{\max}Q\left(s_{k},a_{k}\right). \end{array}$$

There exist two neural networks in the deep *Q* learning algorithm, whose structures are the same and can be called as the critic neural network and target neural network. The parameters of the critic neural network are updated based on temporal-difference learning. The output of the critic neural network is defined as *Q*(*s*_*k*_, *a*_*k*_, *ω*) and the output of the target neural network is defined as *Q*(*s*_*k*_, *a*_*k*_, *ω*^−^). Therefore, the parameters of the critic neural network are updated based on the equation as follows:

(51) $$\begin{array}{c} Q\left(s_{k},a_{k},\omega^{-}\right)=L_{r}\left[R+\gamma_{s}\max_{a^{\prime }}\left(Q\left(s^{\prime },a^{\prime },\omega^{-}\right)\right)\right]+\left(1-L_{r}\right)Q\left(s_{k},a_{k},\omega \right), \end{array}$$

where *L*_*r*_ is the learning rate, *γ*_*s*_ denotes the discount factor, *R* represents the reward of state transition from *s*_*k*_ to *s*′ through action *a*_*k*_, and max_*a*′_(*Q*(*s*′, *a*′, *ω*^−^)) stands for the maximum Q value of the target neural network.

It can be inferred that the reward function has an important influence on the final performance. The design of traditional deep *Q* learning mainly depends on the experience of designers, which can not achieve optimal performance and will improve the computational complexity. In this study, the reward function is applied to design the reward function. The input value of fuzzy reward function can be divided into five categories, which can be described as VB, B, N, G, and VG. The five categories represent very bad, bad, normal, good, and very good. In this study, it is supposed that there are four followers. Therefore, the inputs of the fuzzy reward system are set to be |*e*_1_|, |*e*_2_|, |*e*_3_|, and |*e*_4_|. It can be inferred that each fuzzy set includes 25 rules, and the total number of the fuzzy rules is 75. The output of the fuzzy reward function is limited in the interval [−1,0), and the defuzzifier of the fuzzy reward function is defined as

(52) $$\begin{array}{c} f\left(z\right)=\frac{\sum_{p=1}^{25}C^{i}\prod_{q=1}^{m}s_{q}^{p}\left(z_{q}\right)}{\sum_{p=1}^{25}\prod_{q=1}^{m}s_{q}^{p}\left(z_{q}\right)}. \end{array}$$

Based on the statement above, the learning-based consensus protocol design algorithm can be summarized as follows:

**Remark 2 .**

The FDQN algorithm proposed in this study can improve the transient convergence performance of MASs. The output of the deep *Q* network is supposed to be variation of parameters of consensus protocol. As well known, the design of reward function in the traditional method depends of the experience of the designers. To overcome the problem, the fuzzy reward function is developed to improve the learning efficiency in this study.

## 5. Numerical Example

In this section, an example is provided to illustrate the effectiveness of the method. The model of MASs is constructed as follows:

(53) $$\begin{array}{c} A=\left[\begin{array}{cc} -1\cdot 3 & -1\cdot 5\\ 1\cdot 3 & 1\cdot 2 \end{array}\right],\\ B=\left[\begin{array}{c} 1\cdot 2\\ 1\cdot 5 \end{array}\right],\\ C=\left[\begin{array}{cc} 1\cdot 1 & 0\cdot 9 \end{array}\right],\\ D=\left[\begin{array}{cc} 0\cdot 13 & 0\cdot 12 \end{array}\right]. \end{array}$$

The external disturbance is

(54) $$\begin{array}{c} d\left(k\right)=0\cdot 1e^{-0.2k}\cos\left(0\cdot 4k\right). \end{array}$$

**Figure 1:**
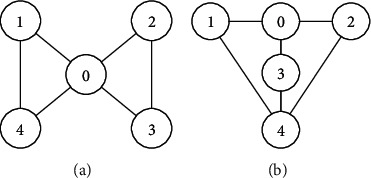
Switching topologies of MASs. (a) Interacting topology *𝒢*_1_. (b) Interacting topology *𝒢*_2_.

The switching topologies are shown in Figure 1. Then, we can obtain the Laplace matrices as follows:

(55) $$\begin{array}{c} L_{1}=\left[\begin{array}{cccc} 1 & 0 & 0 & -1\\ 0 & 1 & -1 & 0\\ 0 & -1 & 1 & 0\\ -1 & 0 & 0 & 1 \end{array}\right],\\ L_{2}=\left[\begin{array}{cccc} 1 & 0 & 0 & -1\\ 0 & 1 & 0 & -1\\ 0 & 0 & 1 & -1\\ -1 & -1 & -1 & 3 \end{array}\right]. \end{array}$$

The parameters of switching topologies are given as follows:

(56) $$\begin{array}{c} \left\{\begin{array}{c} a_{1}=1\cdot 01\\ a_{2}=1\cdot 11\\ a_{3}=1\cdot 08 \end{array}\right),\left\{\begin{array}{c} \mu_{1}=1\cdot 25\\ \mu_{2}=1\cdot 31\\ \mu_{3}=1\cdot 38 \end{array}\right),\left\{\begin{array}{c} \delta_{1}=0\cdot 51\\ \delta_{2}=0\cdot 52\\ \delta_{3}=0\cdot 53 \end{array}\right). \end{array}$$

Therefore, we can obtain MDADT according to (25).

(57) $$\begin{array}{c} \tau_{1}=0\cdot 3128,\;\tau_{2}=0\cdot 3679,\;\tau_{3}=0\cdot 4266. \end{array}$$

It is well known that the ADT method can be viewed as a special case of the MDADT method. Therefore, it can be inferred that *τ*_*a*_=max{*τ*_*ai*_}=0 · 4266. It is obvious that tighter bounds on dwell time and less conservative results can be obtained. Then, we define the attenuation performance index *γ*=0 · 9, and we can obtain the parameters of consensus protocol based on Theorem 2.

**Figure 2:**
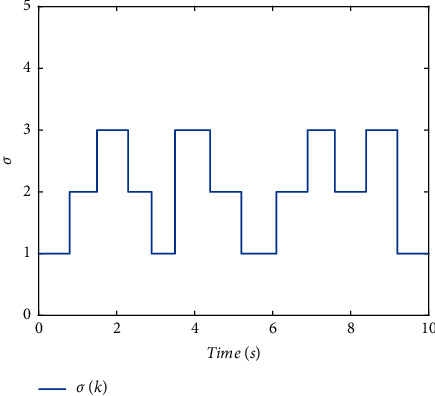
The switching logic.

**Figure 3:**
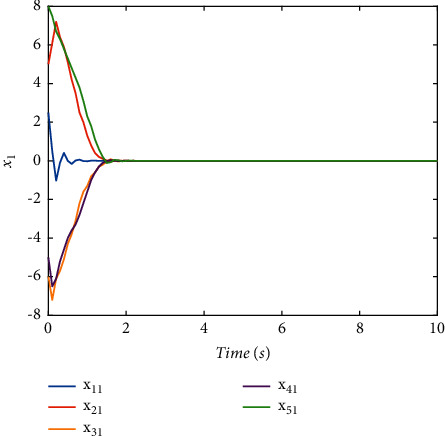
The state response of (*x*)_1_ under the ADT method.

**Figure 4:**
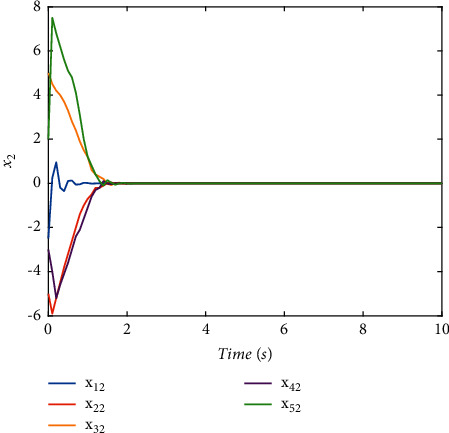
The state response of (*x*)_2_ under the ADT method.

**Figure 5:**
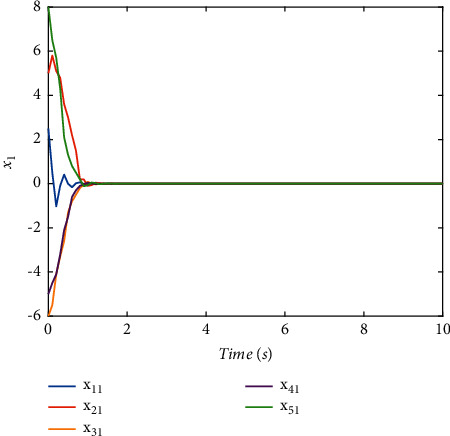
The state response of (*x*)_1_ under the MDADT method.

**Figure 6:**
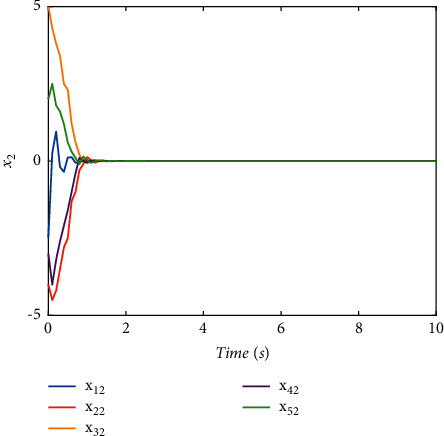
The state response of (*x*)_2_ under the MDADT method.

The switching logic is shown in Figure 2. In order to illustrate the effectiveness and superiority of the proposed method, the traditional ADT method and MDADT method are given as comparisons. From the statement above, we have realized that MDADT can obtain tighter bounds and less conservative results. Moreover, the comparisons of state response of the ADT method and MDADT method are shown in Figures 3–6. The state responses of MASs with ADT switching topologies are shown in Figures 3-4. The state responses of MASs with MDADT switching topologies are shown in Figures 5-6. We can see that the transient performance of the ADT method is better than that of the MDADT method because the different characteristics of subsystems are taken into consideration, which will no doubt improve the design flexibility and make it more applicable for practical conditions.

**Figure 7:**
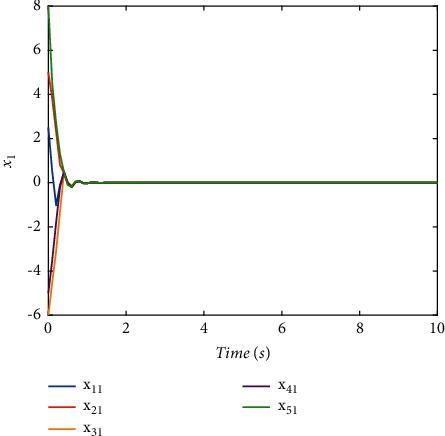
The state response of (*x*)_1_ with the proposed method.

**Figure 8:**
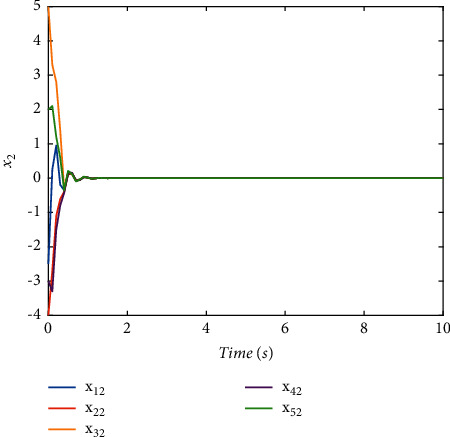
The state response of (*x*)_2_ with the proposed method.

**Figure 9:**
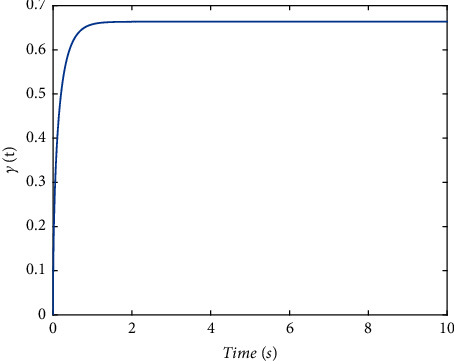
The response of attenuation performance index.

**Figure 10:**
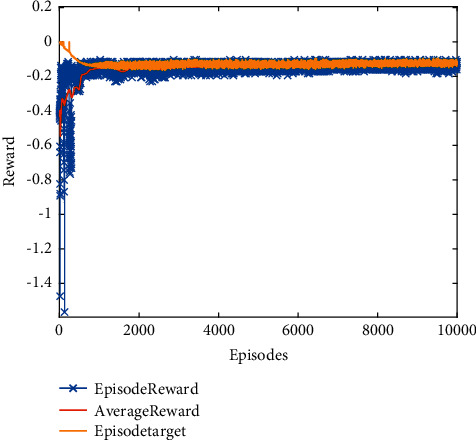
The response of episodes reward.

**Figure 11:**
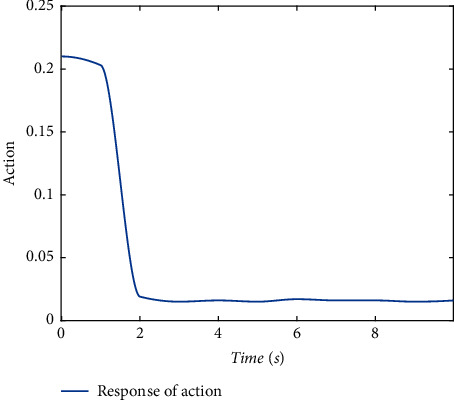
The response of the action.

Validate the superiority of the proposed method. The state response of the proposed method is shown in Figures 7–11. The state responses of the proposed method are shown in Figures 7-8. We can conclude that the transient performance can be improved by the aid of fuzzy deep *Q* learning. The advantages of the traditional method and intelligent method are combined. Compared with the traditional method, the transient performance can be improved, and compared with the intelligent method, stability and training efficiency can be guaranteed. The attenuation performance index is shown in Figure 9, from which we can see that the robustness of the proposed is ensured. The episodes reward response is shown in Figure 10, and we can see that the reward function of the fuzzy deep *Q* learning algorithm can converge to the neighbor of the origin, which demonstrates the effectiveness of the algorithm in this study. In addition, the response of the action is shown in Figure 11, from which we can see that the learning-based consensus protocol is provided to compensate the additional input caused by the uncertainties.

Based on the statement above, we can conclude that the convergence, robustness, and prescribed attenuation performance index are guaranteed. The less conservative results and tighter bounds on dwell time can be obtained by the MDADT method. The transient performance of the system can be improved based on the fuzzy deep *Q* learning algorithm. It is worth mentioning that the traditional robust cannot make comprised of robustness and transient performance, and the intelligent method always cannot guarantee convergence. By employing the proposed method, convergence, robustness, and transient performance are guaranteed simultaneously.

## 6. Conclusions

The problem of intelligent *L*_2_-*L*_∞_ consensus design for MASs under switching topologies is investigated in this study. The switching topologies of MASs are modeled as switched system theory by employing linear transformation. Then, the problem of consensus protocol design can be converted to the problem of *L*_2_-*L*_∞_ control. To ensure the convergence, robustness, and transient performance simultaneously, the proposed consensus protocol is composed of dynamics-based consensus protocol and learning-based consensus protocol, which provides baseline and compensation of uncertainties. The baseline of consensus protocol is obtained by dynamics-based consensus protocol, which is provided based on the MDADT method and MLF method. The scheduling interval of learning-based protocol is given by nonfragile control theory. Then, the learning-based consensus protocol is proposed based on the fuzzy deep *Q* learning algorithm to improve the transient performance and achieve optimal policy, where the fuzzy reward function is introduced to improve the learning efficiency.

## Figures and Tables

**Algorithm 1:**
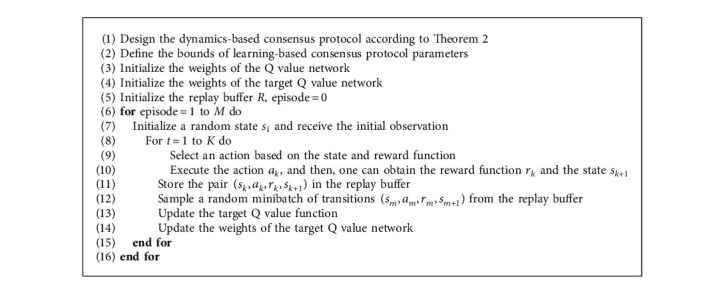
Learning-based control protocol design based on FDQN.

## Data Availability

The data used to support the findings of this study are included within the article.
